# Pervasive mixed infections and recurrent begomovirus co-detection in symptomatic pumpkin plants from Guangxi, China

**DOI:** 10.3389/fmicb.2026.1857304

**Published:** 2026-06-12

**Authors:** Baoling Chen, Yanxia Zhou, Man Zhang, Jianhui Zhou, Zhenglin Wan, Wenjun Liu

**Affiliations:** Institute of Vegetables, Guangxi Academy of Agricultural Sciences, Nanning, Guangxi, China

**Keywords:** begomovirus, cucurbit viruses, mixed infection, pumpkin, ToLCNDV

## Abstract

**Introduction:**

Pumpkin production in Guangxi, China, is increasingly affected by complex viral diseases, but the regional viral spectrum and mixed-infection structure in symptomatic pumpkin plants remain insufficiently characterized.

**Methods:**

We analyzed 289 symptomatic pumpkin samples collected across Guangxi during 2022-2025 using small-RNA sequencing for virus discovery and targeted PCR/RT-PCR assays for individual-sample confirmation. Virus prevalence, mixed-infection profiles, regional and host-associated differences, and virus-virus and symptom-virus associations were evaluated.

**Results:**

Small-RNA sequencing identified 15 candidate virus species, and targeted assays confirmed 11 viruses. Among the 289 samples, 284 (98.27%) were positive for at least one confirmed virus and 279 (96.54%) carried two or more viruses. The dataset was dominated by PuYmMV, SLCPHV, TLCCNV, ToLCNDV, and SLCCNV, which were detected in 87.89%, 87.89%, 83.39%, 78.89%, and 68.86% of samples, respectively. The most frequent infection profile was simultaneous detection of these five begomovirus species, occurring in 35.64% of samples. After Benjamini-Hochberg correction, ZTMV and PRSV were enriched in northern Guangxi, whereas TLCCNV and ToLCNDV were enriched in southern Guangxi. Host-species differences were not significant after correction. Pairwise analysis revealed a strong begomovirus-rich co-detection module, whereas symptom-virus associations were comparatively weak.

**Discussion:**

Symptomatic pumpkin viral disease in Guangxi is characterized by broad virus diversity, pervasive mixed infection, and recurrent begomovirus-rich co-detection. These findings provide a regional baseline for multiplex diagnosis, begomovirus-oriented surveillance, and future plant-vector-reservoir studies in pumpkin production systems.

## 1 Introduction

Viral diseases are major biological constraints on cucurbit production because they reduce yield, fruit quality, and marketability, and they are often difficult to manage once inoculum sources and vectors are established in the field (Lecoq and Desbiez, 2012; Navas-Castillo et al., 2011; Radouane et al., 2021). Cucurbit crops are affected by a wide range of viruses belonging to different taxonomic groups, including potyviruses, poleroviruses, criniviruses, tobamoviruses, and begomoviruses, and the dominant viral spectrum can vary substantially among production regions and cropping systems (Lecoq and Desbiez, 2012; Navas-Castillo et al., 2011; Li et al., 2022). Another important feature of cucurbit virology is the frequent occurrence of mixed infection. In naturally infected plants, multiple viruses are often detected in the same host, and these mixed infections can alter symptom expression, virus accumulation, and the interpretation of field phenotypes (Moreno and López-Moya, 2020; Sadras et al., 2024; Moya-Ruiz et al., 2024; Sánchez-Tovar et al., 2025). As a result, symptom-based diagnosis alone is often insufficient in cucurbit fields where several viruses circulate simultaneously (Moreno and López-Moya, 2020; Adachi-Fukunaga and Tomitaka, 2025).

Recent regional surveys have shown that cucurbit virus composition is highly context dependent. In Guangdong, southern China, small-RNA-based surveillance identified multiple viruses in cucurbit crops, with Papaya ringspot virus (PRSV), Zucchini tigre mosaic virus (ZTMV), Zucchini yellow mosaic virus (ZYMV), and Watermelon silver mottle virus (WSMoV) among the major detected viruses, indicating a comparatively large contribution of RNA viruses to the local disease complex (Li et al., 2022). In Oklahoma, surveys also identified systems dominated by potyviruses such as PRSV-W, Watermelon mosaic virus (WMV), and ZYMV (Khanal et al., 2021). In the Spanish Mediterranean area, recent work likewise documented substantial cucurbit virus diversity across crops and locations rather than a single overwhelmingly dominant pattern (López-Martín et al., 2024). By contrast, begomovirus-dominated cucurbit epidemics with frequent mixed infections have been reported from Tamil Nadu and Uttar Pradesh, where begomoviruses represented a major component of symptomatic cucurbit infections (Nagendran et al., 2017; Kumari et al., 2021). These comparisons indicate that regional surveys remain necessary not only to determine which viruses are present, but also to define whether local disease burden is structured mainly by begomoviruses, potyviruses, or a broader mixed assemblage (Lecoq and Desbiez, 2012; Li et al., 2022; Khanal et al., 2021; López-Martín et al., 2024; Nagendran et al., 2017; Kumari et al., 2021).

Among the virus groups currently reshaping cucurbit disease risk, whitefly-transmitted begomoviruses have received increasing attention because of their efficient spread, broad host ranges, and persistence in intensive vegetable production systems (Navas-Castillo et al., 2011; Fiallo-Olivé et al., 2020; Cai et al., 2023). Their epidemiological importance is closely tied to the biology of Bemisia tabaci, and transmission efficiency can differ among whitefly species or cryptic species (Fiallo-Olivé et al., 2020; Shan et al., 2025; Wang et al., 2025). Within this broader begomovirus context, Begomovirus solanumdelhiense (Tomato leaf curl New Delhi virus, ToLCNDV) has emerged as a high-concern cucurbit pathogen because of its broad host range, expanding distribution, and increasing economic relevance (Moriones et al., 2017; Cai et al., 2023; Vo et al., 2025). In China, ToLCNDV has already been reported from multiple cucurbit hosts, indicating that it should be considered part of the current cucurbit disease landscape rather than an isolated record (Gu et al., 2023; Zeng et al., 2023). At the same time, ToLCNDV should not be interpreted in isolation in field systems where several begomoviruses co-circulate, because disease pressure may reflect a broader begomovirus complex rather than a single focal virus (Moriones et al., 2017; Cai et al., 2023; Vo et al., 2025).

Guangxi is an important cucurbit-producing region in southern China, and its warm climate, prolonged cropping periods, and intensive vegetable production may favor repeated virus circulation and transmission. However, despite the agricultural importance of pumpkin in Guangxi, Guangxi-wide information on the viruses associated with symptomatic pumpkin plants has remained limited. In particular, three questions have remained insufficiently addressed: which virus species are associated with symptomatic pumpkin plants at the regional scale, how common mixed infections are and which combinations dominate, and whether the Guangxi system more closely resembles RNA-virus-rich cucurbit surveys reported from some regions or begomovirus-rich systems reported from others (Li et al., 2022; Khanal et al., 2021; López-Martín et al., 2024; Nagendran et al., 2017; Kumari et al., 2021). These questions are practically relevant because local virus spectra determine which molecular assays are most informative, which viruses should be prioritized in breeding and surveillance, and whether single-virus diagnostics are likely to underestimate field virus burden (Moreno and López-Moya, 2020; Adachi-Fukunaga and Tomitaka, 2025; Koeda et al., 2024; Schafleitner et al., 2024).

This study aimed to define the viral community associated with symptomatic pumpkin plants in Guangxi at the regional scale. Specifically, this study examined the virus species associated with symptomatic pumpkin plants in Guangxi, the frequency of mixed infections and the most recurrent infection profiles, the presence of regional and host-associated differences in the plant dataset, and whether virus–virus co-detection patterns were stronger than symptom-linked associations under field conditions. By addressing these questions, this study provides a regional baseline for multiplex diagnosis, begomovirus-oriented surveillance, and subsequent plant–vector–reservoir studies in Guangxi pumpkin production systems.

## 2 Materials and methods

### 2.1 Field surveys and sample collection

**FIGURE 1:**
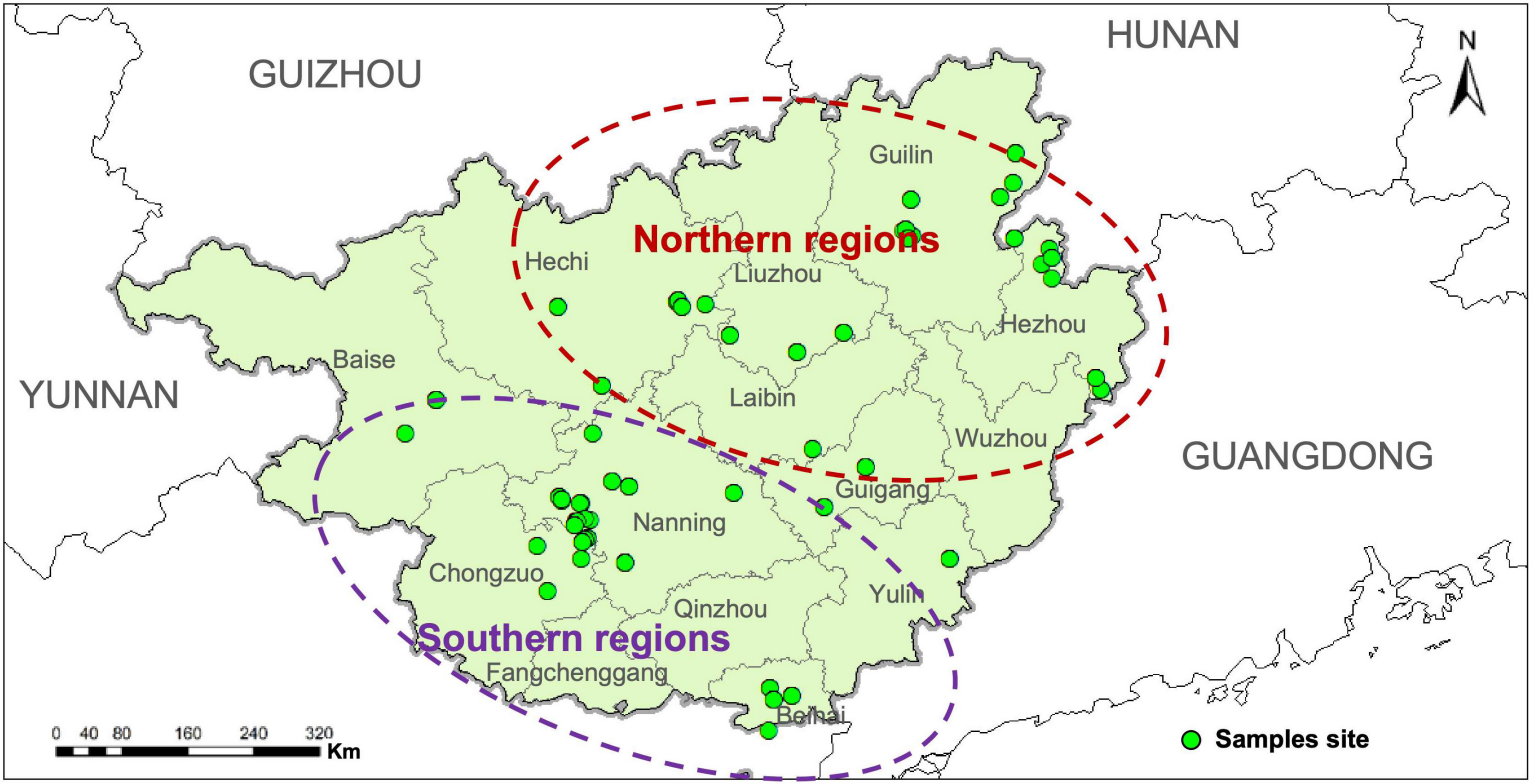
Map of Guangxi Zhuang Autonomous Region showing the 14 prefecture-level cities where symptomatic pumpkin samples were collected. Sampling sites are indicated on the map. The northern Guangxi and southern Guangxi groups used for regional comparison are also shown.

Following previous cucurbit virus survey frameworks (Li et al., 2022; Khanal et al., 2021; López-Martín et al., 2024), field surveys were conducted in major pumpkin-producing areas of Guangxi Zhuang Autonomous Region, China, from September 2022 to October 2025. Symptomatic pumpkin plants were sampled across 14 prefecture-level cities, including Nanning, Chongzuo, Beihai, Fangchenggang, Qinzhou, Yulin, Guigang, Laibin, Liuzhou, Guilin, Hezhou, Hechi, Baise, and Wuzhou (Figure 1 and Supplementary Table 1). A total of 289 leaf samples were collected from individual plants showing virus-like symptoms, including chlorosis/yellowing, leaf deformation, blistering, mosaic, leaf curling, and stunting.

The sampled plants represented the three main cultivated pumpkin species in the region, namely *Cucurbita moschata*, *C. maxima*, and *C. pepo*. For each sample, metadata including sampling location, collection date, host species, and observed symptoms were recorded in the field. Leaf tissues were dried immediately with silica gel after sampling and stored at −80 °C until nucleic acid extraction.

### 2.2 Small-RNA sequencing for virus discovery

For initial virus discovery, small-RNA (sRNA) sequencing was performed on representative symptomatic pumpkin samples covering different sampling regions and major symptom phenotypes observed during field surveys, because sRNA-based sequencing has been widely used for plant virus discovery and surveillance (Kreuze et al., 2009; Li et al., 2022). Total RNA was extracted from leaf tissues using TRIzol^®^ reagent (Invitrogen, Carlsbad, CA, United States) according to the manufacturer’s instructions. RNA quality and concentration were assessed by spectrophotometry and agarose gel electrophoresis prior to library construction.

For broad virus discovery, four pooled sRNA libraries were constructed from representative samples grouped according to sampling region and major symptom type. Each pool contained equal amounts of total RNA from multiple symptomatic samples showing comparable symptom phenotypes within the same or similar geographical background. To complement pooled discovery and verify virus occurrence at the individual-sample level, 14 representative samples covering different regions and symptom phenotypes were additionally subjected to independent sRNA sequencing.

Pooled sRNA libraries were sequenced by Novogene Co., Ltd., (Beijing, China) on an Illumina NovaSeq 6000 platform. The 14 individual sRNA libraries were prepared using the NEBNext^®^ Multiplex Small RNA Library Prep Kit (New England Biolabs, Ipswich, MA, United States), size-selected for 18–35 nt fragments, and sequenced on an Illumina HiSeq 2500 platform with 50-bp single-end reads at Biomarker Technologies Corporation (Beijing, China).

### 2.3 Bioinformatic identification of candidate viruses

Raw sRNA reads were processed using fastp (v0.20.0) to remove adapter sequences, low-quality reads, and contaminating sequences (Chen et al., 2018). Reads shorter than 18 nt or longer than 35 nt were discarded. Clean reads were assembled *de novo* using Velvet (v1.2.10) with an optimized k-mer size of 17 (Zerbino and Birney, 2008).

The resulting contigs were compared against viral reference sequences in the NCBI nucleotide database using BLASTn with an E-value cutoff of 1 × 10^–5^ (Altschul et al., 1990). Candidate viruses were assigned on the basis of significant sequence similarity to known plant viruses. For the 14 individually sequenced samples, clean sRNA reads were further mapped to reference virus genomes using Bowtie2 (v2.4.5), and viral small interfering RNA abundance was normalized as reads per million (RPM) of total clean sRNA reads (Langmead and Salzberg, 2012).

Viruses detected by sRNA sequencing were regarded as candidate viruses and were subsequently subjected to targeted molecular validation in individual field samples. Only viruses supported by targeted amplification in individual samples were treated as confirmed viruses in downstream analyses.

### 2.4 Targeted molecular detection and sequence confirmation

Based on the sRNA sequencing results, virus-specific primers were designed to amplify conserved genomic regions of the detected candidate viruses (Supplementary Table 4). Targeted molecular screening was then performed for all 289 field samples to validate virus occurrence in individual plants, following the common strategy of high-throughput virus discovery followed by targeted PCR/RT-PCR confirmation (Kreuze et al., 2009; Li et al., 2022).

For RNA viruses, total RNA was extracted from approximately 100 mg of silica-gel-dried leaf tissue using TRIzol^®^ reagent (Invitrogen, Carlsbad, CA, United States) following the manufacturer’s instructions. One-step RT-PCR assays were performed using the PrimeScript One Step RT-PCR Kit Ver.2 (TaKaRa Bio Inc., Kusatsu, Shiga, Japan). Approximately 0.5 μg of total RNA was used as template in each 25 μL reaction, and virus-specific forward and reverse primers listed in Supplementary Table 4 were included directly in the one-step reactions. No separate oligo(dT)-primed cDNA synthesis was used; therefore, reverse transcription did not depend on the presence of a poly(A) tail, which is particularly important for detecting non-polyadenylated RNA viruses such as crinivirus candidates. The thermal program consisted of reverse transcription at 50 °C for 30 min, initial denaturation at 94 °C for 2 min, followed by 35 cycles of 94 °C for 30 s, primer-specific annealing at 52 °C–58 °C for 30 s, and 72 °C for 1 min per kb, with a final extension at 72 °C for 10 min.

For begomoviruses, total DNA was extracted from an independent aliquot of the same dried leaf sample using the DP305 Plant Genomic DNA Kit (Tiangen Biotech, Beijing, China) according to the manufacturer’s instructions. PCR amplification was performed with begomovirus-specific primers targeting conserved genomic regions. Each PCR reaction was carried out in a 25 μL volume containing 12.5 μL of 2 × Taq PCR Master Mix, 1.0 μL of forward primer (10 μM), 1.0 μL of reverse primer (10 μM), 1.0–2.0 μL of template DNA, and nuclease-free water to the final volume. The PCR program consisted of an initial denaturation at 95 °C for 3 min, followed by 35 cycles of 95 °C for 30 s, primer-specific annealing at 52 °C–58°C for 30 s, and 72 °C for 45–60 s, with a final extension at 72 °C for 10 min. Annealing temperatures were optimized according to primer pairs and are summarized in Supplementary Table 4.

Polymerase chain reaction (PCR) and reverse-transcription PCR (RT-PCR) products were separated on 1.5% agarose gels and visualized under UV illumination. Amplicons of the expected size were purified, cloned into the pMD19-T vector (TaKaRa Bio Inc., Kusatsu, Shiga, Japan), and subjected to Sanger sequencing for identity confirmation. The obtained diagnostic sequences were compared with GenBank reference sequences using BLASTn. Only viruses supported by targeted amplification and sequence confirmation in individual samples were treated as confirmed viruses in subsequent analyses. Because these amplicons represented short conserved diagnostic regions, they were used for molecular confirmation rather than for formal species demarcation, isolate-level evolutionary inference, or phylogenetic tree construction. For begomoviruses, detection was interpreted as correspondence to known virus species based on specific amplification and sequence confirmation; formal species or strain characterization would require complete DNA-A genome sequences.

### 2.5 Symptom classification and mixed-infection profiling

Symptoms observed in the field were classified into six categories: chlorosis/yellowing, leaf deformation, blistering, mosaic, leaf curling, and stunting. Because individual plants frequently exhibited more than one symptom and mixed viral infections can complicate symptom interpretation, symptom data were recorded as non-mutually exclusive binary variables for each category (Moreno and López-Moya, 2020; Sadras et al., 2024).

For each sample, mixed-infection complexity was defined as the number of confirmed viruses detected in that individual plant. Infection profiles were summarized as the unique combinations of confirmed viruses observed across the 289 samples, and the frequencies of recurrent profiles were calculated.

### 2.6 Regional and host-group comparisons

To examine large-scale spatial variation in virus occurrence, samples were grouped into southern Guangxi and northern Guangxi according to the sampling framework used in this study. The southern Guangxi group included Nanning, Chongzuo, and Beihai, whereas the northern Guangxi group included Guilin, Hezhou, Hechi, and Liuzhou. Cities outside these two groups were not included in the formal north–south comparison because of limited sample numbers or lack of balanced representation for inferential analysis.

For host-related comparisons, samples were assigned to *Cucurbita moschata*, *C. maxima*, or *C. pepo*. One interspecific hybrid accession was excluded from the formal host-species comparison.

### 2.7 Statistical analysis

All statistical analyses were performed in IBM SPSS Statistics v29. Virus detection rates were calculated as the percentage of samples positive for each confirmed virus among the 289 sampled plants. Symptom frequencies and mixed-infection complexity classes were summarized descriptively.

Differences in virus detection rates between regions and among host species were evaluated using Pearson’s chi-square test or Fisher’s exact test where appropriate. Because multiple viruses were tested in parallel, raw *P*-values from regional and host-species comparisons were adjusted separately using the Benjamini–Hochberg false discovery rate procedure (Benjamini and Hochberg, 1995). Adjusted q values < 0.05 were considered statistically significant.

Pairwise virus–virus and symptom–virus associations were evaluated using the phi (φ) coefficient based on binary presence/absence matrices, a standard measure for association between binary variables. For symptom–virus analyses, each symptom category was treated as an independent binary variable because symptom categories were not mutually exclusive. Raw *P*-values from association analyses were also adjusted using the Benjamini–Hochberg procedure (Benjamini and Hochberg, 1995), and associations with q < 0.05 were regarded as significant.

Association heatmaps included both positive and negative φ values to show the direction and magnitude of pairwise relationships. For network visualization, only positive associations with q < 0.05 and φ ≥ 0.25 were retained as edges. Negative associations were summarized in the heatmaps only. Network visualization was performed in Cytoscape v3.10.4 (Shannon et al., 2003).

## 3 Results

### 3.1 Symptom composition of symptomatic pumpkin samples in Guangxi

**FIGURE 2:**
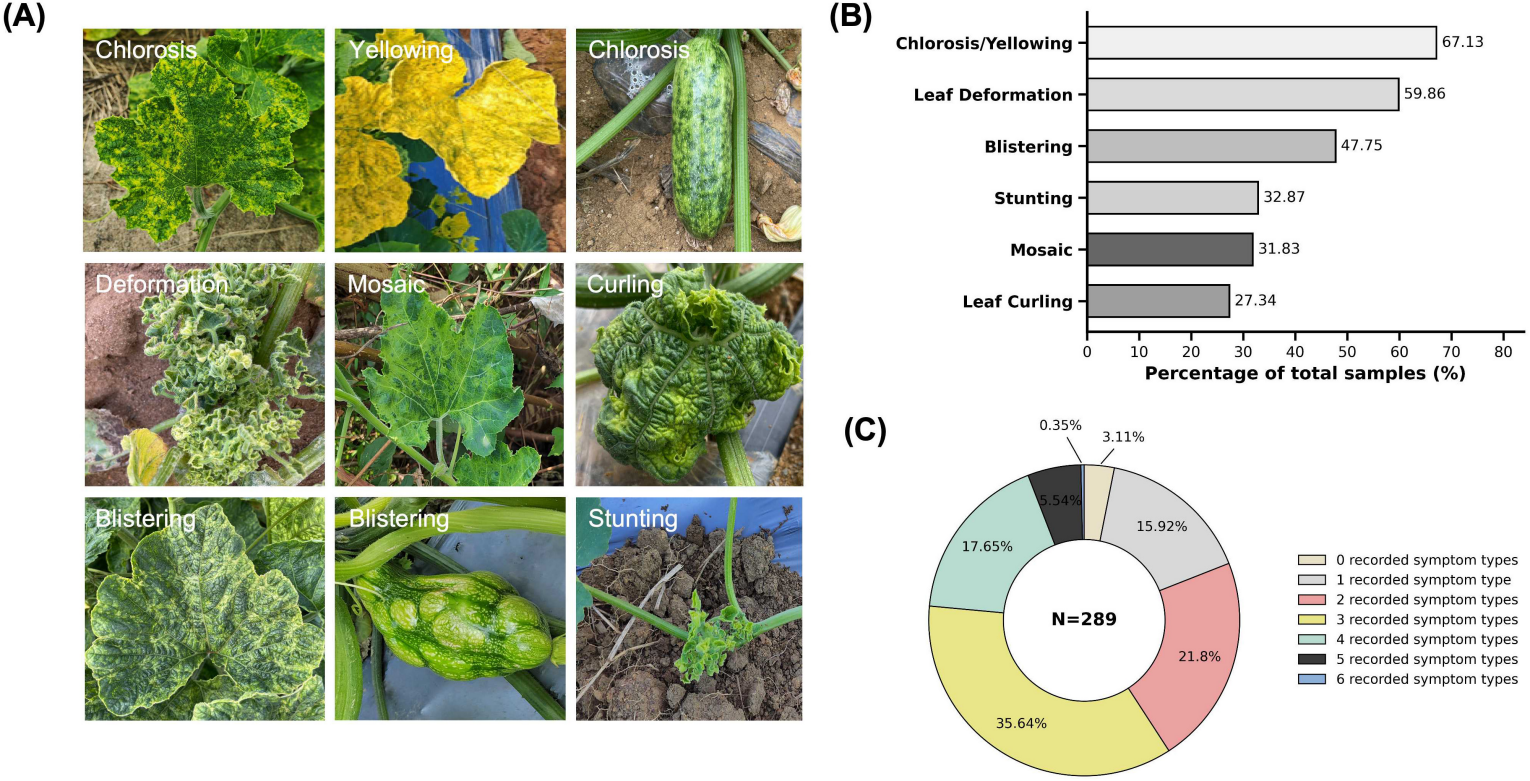
Symptom composition and symptom complexity in symptomatic pumpkin samples collected in Guangxi, China. **(A)** Representative symptoms observed in field-collected pumpkin plants in Guangxi. **(B)** Frequencies of six symptom categories recorded in 289 symptomatic samples. Values represent the percentage of total samples exhibiting each symptom category. **(C)** Distribution of symptom complexity, expressed as the number of symptom categories recorded per sample.

Field surveys conducted across Guangxi from 2022 to 2025 yielded 289 symptomatic pumpkin samples, and representative symptoms are shown in Figure 2A. When symptoms were classified into six categories, chlorosis/yellowing was the most frequent symptom (67.13%), followed by leaf deformation (59.86%), blistering (47.75%), stunting (32.87%), mosaic (31.83%), and leaf curling (27.34%) (Figure 2B). Overall, chlorosis/yellowing and deformation were the predominant symptom types in the symptomatic dataset.

Most plants displayed more than one symptom category. The three-symptom class was the most common (35.64%), followed by the two-symptom (21.80%) and four-symptom (17.65%) classes (Figure 2C). Samples with two to four symptom categories together accounted for 75.09% of the dataset, indicating that symptom expression in the field was usually composite rather than isolated.

### 3.2 Candidate and confirmed viruses detected in symptomatic pumpkin plants

Small-RNA sequencing identified contigs corresponding to 15 candidate virus species in symptomatic pumpkin samples from Guangxi (Supplementary Table 2). These candidates included Zucchini yellow mosaic virus (ZYMV), Watermelon mosaic virus (WMV), Squash leaf curl China virus (SLCCNV), Pumpkin yellow mosaic Malaysia virus (PuYmMV), Squash leaf curl Philippines virus (SLCPHV), Tomato leaf curl China virus (TLCCNV), Tomato leaf curl New Delhi virus (ToLCNDV), Zucchini tigre mosaic virus (ZTMV), Papaya ringspot virus (PRSV), Watermelon silver mottle virus (WSMoV), Squash aphid-borne yellows virus (SABYV), Cucurbit aphid-borne yellows virus (CABYV), Cucurbit yellow stunting disorder virus (CYSDV), Luffa aphid-borne yellows virus (LASYV), and Cucumber green mottle mosaic virus (CGMMV) (Supplementary Table 2). Virus names, taxonomic species names, and abbreviations were standardized according to current ICTV nomenclature principles (Zerbini et al., 2022).

**FIGURE 3:**
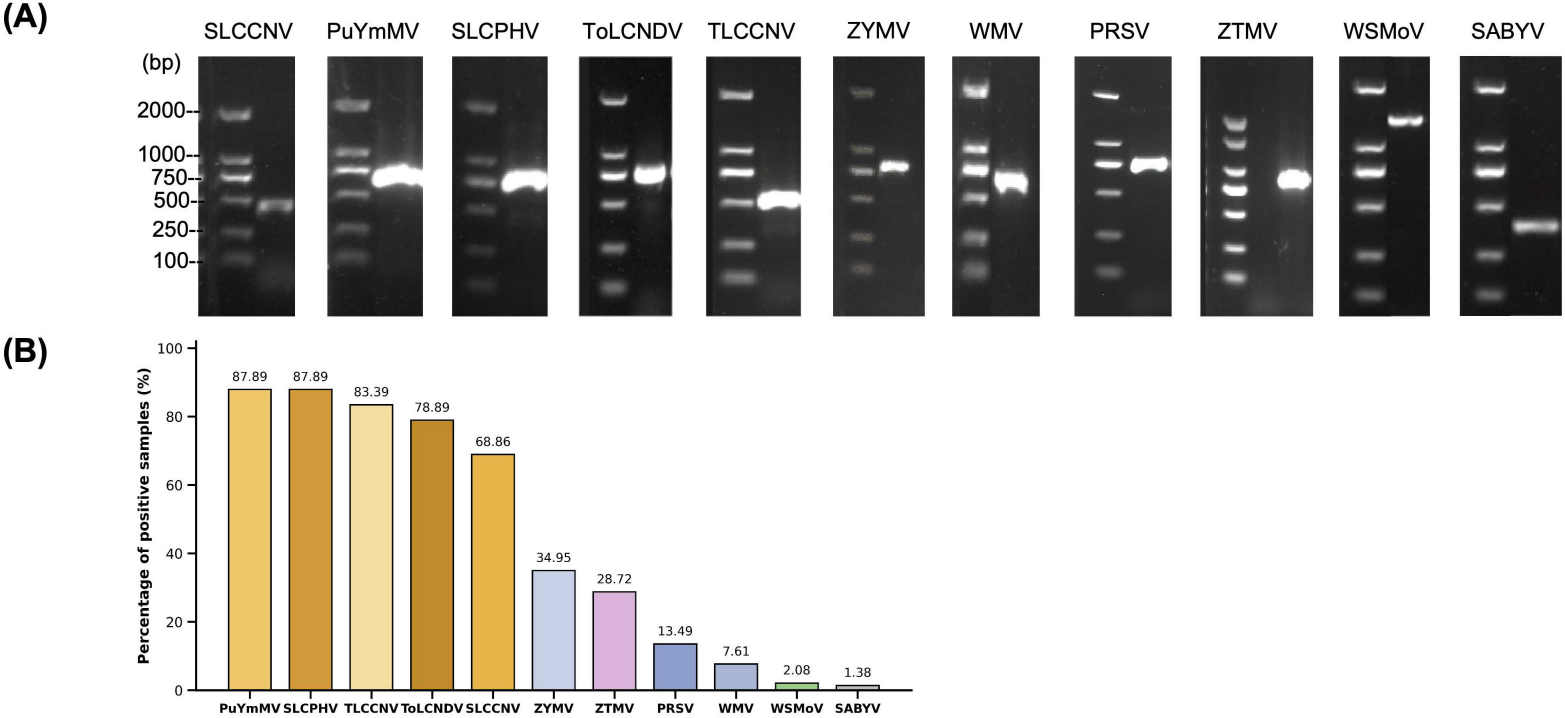
Molecular detection and prevalence of viruses in symptomatic pumpkin samples from Guangxi, China. **(A)** Representative agarose gel electrophoresis images of diagnostic amplicons obtained for selected viruses. **(B)** Detection rates of the 11 confirmed viruses identified by targeted PCR/RT-PCR screening of 289 symptomatic pumpkin samples. Viruses are ordered by descending prevalence. The five most prevalent viruses were PuYmMV, SLCPHV, TLCCNV, ToLCNDV, and SLCCNV. Values above bars indicate detection rates (%).

Targeted PCR/RT-PCR screening of all 289 samples confirmed 11 of these viruses, as illustrated by representative amplicons in Figure 3A, and their taxonomic classification is summarized in Supplementary Table 3. The confirmed-virus dataset was dominated by five begomovirus species. The official ICTV binomial species names of these viruses are provided in Supplementary Table 3; after first definition, abbreviations are used throughout the text for readability. PuYmMV and SLCPHV were each detected in 87.89% of samples, followed by TLCCNV (83.39%), ToLCNDV (78.89%), and SLCCNV (68.86%) (Figure 3B). Among the RNA viruses, ZYMV (34.95%) and ZTMV (28.72%) were the most frequently detected, whereas PRSV (13.49%), WMV (7.61%), WSMoV (2.08%), and SABYV (1.38%) were detected less often (Figure 3B). In total, 284 of the 289 symptomatic samples (98.27%) were positive for at least one of the 11 confirmed viruses, whereas five samples (1.73%) were negative for all targeted assays (Supplementary Table 6). The Sanger-confirmed diagnostic fragments were used to verify amplicon identity by BLASTn comparison, but no new virus species, new isolates, or evolutionary relationships were inferred from these short conserved fragments.

### 3.3 Mixed-infection profiles in symptomatic pumpkin samples

**FIGURE 4:**
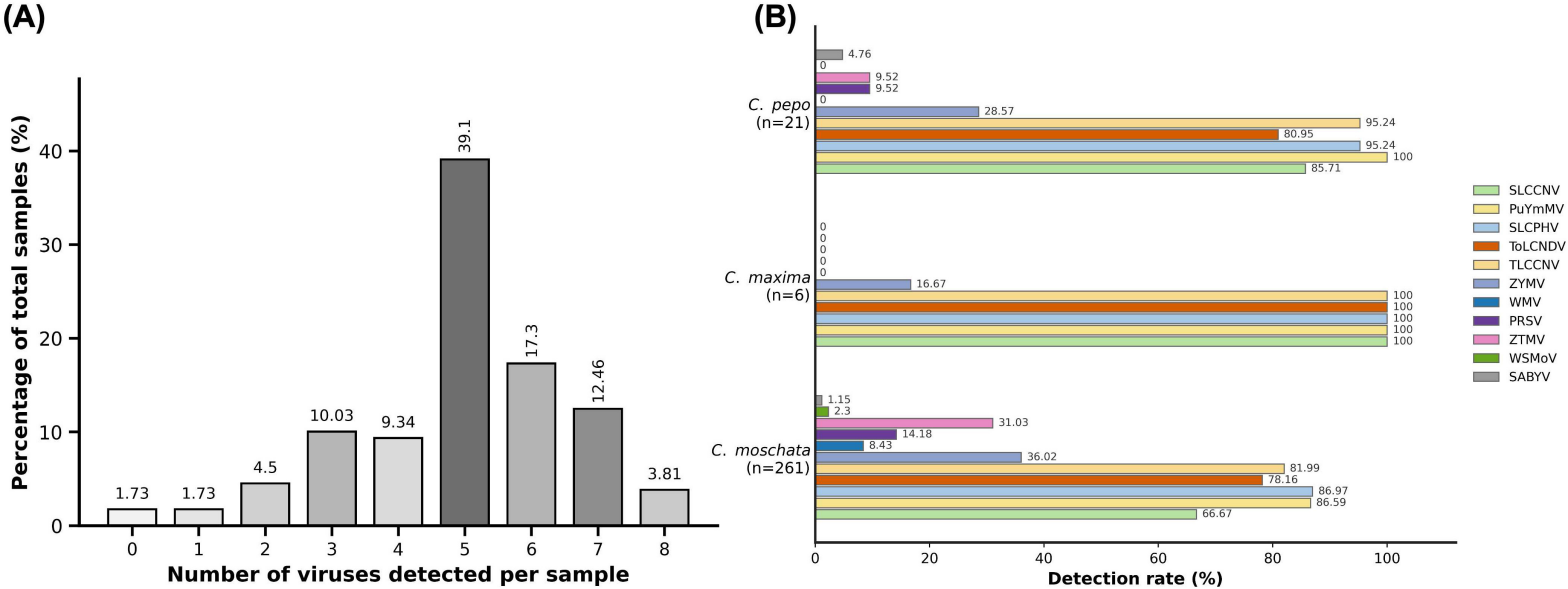
Mixed-infection complexity and host-associated virus detection patterns in symptomatic pumpkin samples from Guangxi, China. **(A)** Distribution of infection complexity, expressed as the number of confirmed viruses detected per sample. Values above bars indicate the percentage of total samples in each complexity class. **(B)** Detection rates of the 11 confirmed viruses across the three cultivated pumpkin species, *Cucurbita moschata* (*n* = 261), *C. maxima* (*n* = 6), and *C. pepo* (*n* = 21). Values above bars indicate the percentage of samples positive for each virus within each host species.

Mixed infection was a dominant feature of the confirmed-virus dataset. Of the 289 samples, 279 (96.54%) carried two or more confirmed viruses; among virus-positive samples, this proportion was 98.24% (279/284). The most frequent complexity class was five viruses per sample (39.10%), followed by six viruses (17.30%), seven viruses (12.46%), and three viruses (10.03%) (Figure 4A and Supplementary Table 6). Only five samples contained a single confirmed virus, and five samples were negative for all 11 assays (Supplementary Table 6).

At the profile level, the most frequent combination was the five-begomovirus profile SLCCNV + PuYmMV + SLCPHV + TLCCNV + ToLCNDV, which occurred in 103 samples (35.64%) (Supplementary Table 6). The next most frequent profile was the same five-begomovirus combination plus ZYMV, detected in 30 samples (10.38%) (Supplementary Table 6). Additional recurrent profiles included SLCCNV + PuYmMV + SLCPHV + ZTMV + PRSV + TLCCNV + ToLCNDV (14 samples, 4.84%) and ZYMV + SLCCNV + PuYmMV + SLCPHV +ZTMV +TLCCNV + ToLCNDV (13 samples, 4.50%) (Supplementary Table 6). Thus, mixed infections in this dataset were concentrated in a limited number of repeated profiles centered on the dominant begomoviruses.

### 3.4 Regional and host-associated differences in virus detection

Regional comparisons between southern and northern Guangxi showed that several viruses remained significantly different after Benjamini–Hochberg correction for multiple testing (Supplementary Table 5). ZTMV was detected more frequently in northern Guangxi than in southern Guangxi (43.97% vs. 18.50%, q < 0.05) (Supplementary Table 5). PRSV was also enriched in northern Guangxi (20.69% vs. 8.67%, q < 0.05) (Supplementary Table 5). In contrast, TLCCNV and ToLCNDV were more prevalent in southern Guangxi than in northern Guangxi, with detection rates of 89.60% versus 74.14% for TLCCNV and 84.39% versus 70.69% for ToLCNDV, respectively (both q < 0.05) (Supplementary Table 5). Other viruses, including ZYMV, WMV, SLCCNV, PuYmMV, and SLCPHV, did not differ significantly between the two regions after correction (Supplementary Table 5).

Host-species comparisons were restricted to 288 samples assigned to *Cucurbita moschata* (*n* = 261), *C. maxima* (*n* = 6), or *C. pepo* (*n* = 21), excluding one interspecific hybrid accession from the formal comparison (Figure 4B and Supplementary Table 5). Descriptively, begomovirus detection remained high in *C. maxima* and *C. pepo*, whereas WMV, PRSV, and ZTMV were observed mainly in *C. moschata* (Figure 4B and Supplementary Table 5). However, none of the 11 viruses showed a statistically significant host-species difference after Benjamini–Hochberg correction (all q > 0.05) (Supplementary Table 5). These host-related patterns should therefore be interpreted as descriptive frequency differences.

### 3.5 Virus–virus and symptom–virus association patterns

**FIGURE 5:**
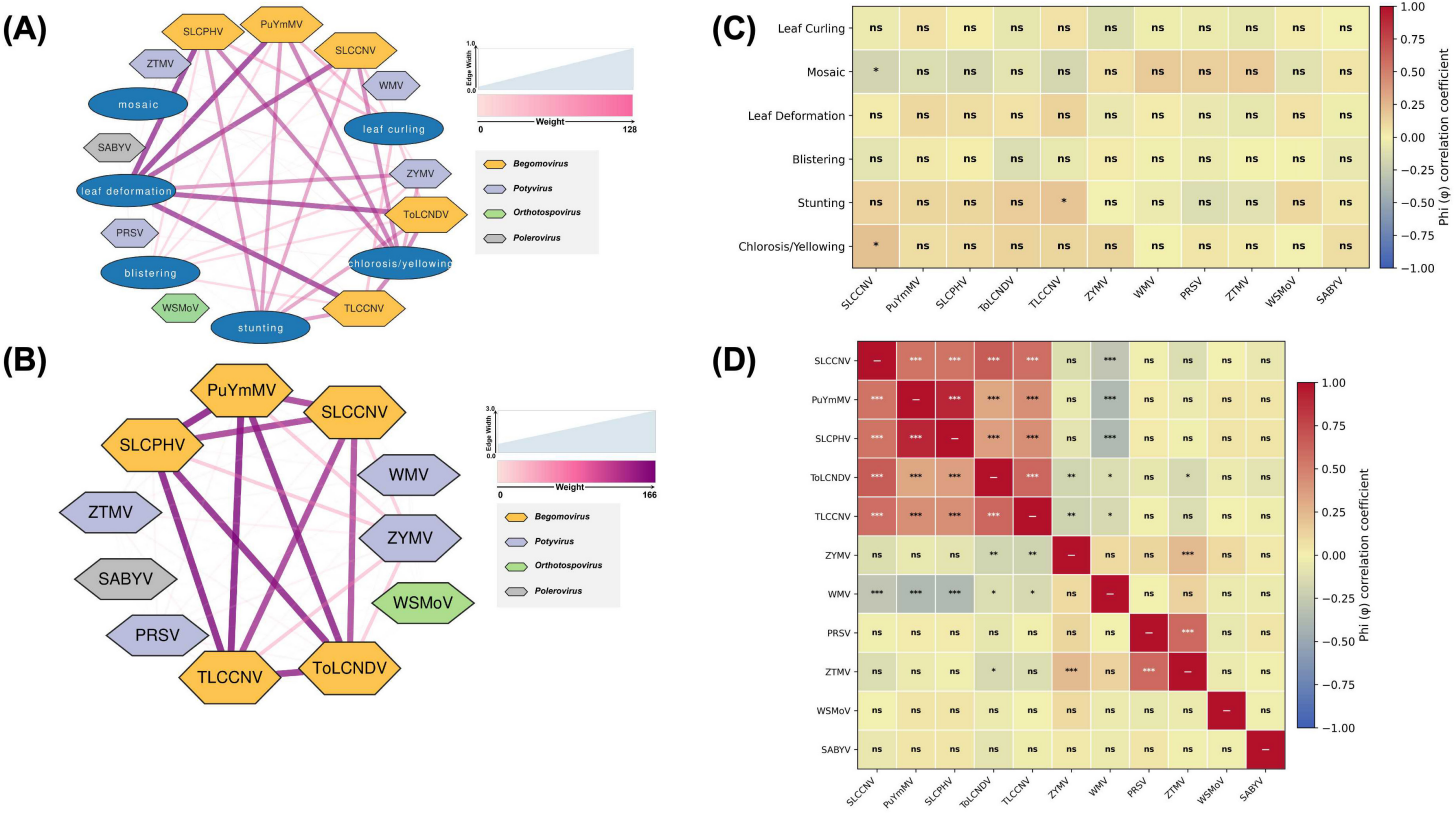
Virus–virus and symptom–virus association patterns in symptomatic pumpkin samples from Guangxi, China. **(A)** Symptom–virus association network showing significant positive associations retained for visualization. **(B)** Virus–virus co-detection network showing significant positive associations retained for visualization. **(C)** Heatmap of symptom–virus phi (φ) coefficients. **(D)** Heatmap of virus–virus phi (φ) coefficients. In panels **(C,D)**, cell color indicates the direction and magnitude of the association, and symbols denote significance levels after multiple-testing correction. Only positive associations with q < 0.05 and φ ≥ 0.25 were displayed in the network panels.

Pairwise virus–virus association analysis identified a strongly connected positive co-detection module among the dominant begomoviruses, as shown in the co-detection network and heatmap (Figures 5B,D and Supplementary Table 7). The strongest association in the matrix was between PuYmMV and SLCPHV (φ = 0.902, q < 0.001) (Figure 5D and Supplementary Table 7). Additional strong positive associations were detected for SLCCNV–ToLCNDV (φ = 0.659, q < 0.001), TLCCNV–ToLCNDV (φ = 0.612, q < 0.001), SLCCNV–TLCCNV (φ = 0.583, q < 0.001), SLCCNV–PuYmMV (φ = 0.552, q < 0.001), and SLCCNV–SLCPHV (φ = 0.552, q < 0.001) (Figure 5D and Supplementary Table 7). Positive associations also linked PuYmMV and SLCPHV separately with TLCCNV and ToLCNDV (φ = 0.328–0.433; all q < 0.001) (Figure 5D and Supplementary Table 7). Together, these results define a recurrent begomovirus-rich co-detection module.

Outside this major module, the most prominent RNA-virus association was between ZTMV and PRSV (φ = 0.600, q < 0.001), and a weaker but still significant positive association was detected between ZYMV and ZTMV (φ = 0.240, q < 0.001) (Figure 5D and Supplementary Table 7). Several significant negative associations were also observed. WMV showed negative correlations with PuYmMV, SLCPHV, SLCCNV, TLCCNV, and ToLCNDV (φ = −0.139 to −0.373; q < 0.05 or q < 0.001), and ZYMV was negatively associated with TLCCNV and ToLCNDV (φ = −0.199 and −0.190, respectively; both q < 0.01) (Figure 5D and Supplementary Table 7). Overall, the association matrix contained both positive and negative structure, with the strongest positive associations occurring within the begomovirus subset.

In contrast, symptom–virus associations were much more limited after correction for multiple testing, as shown in the symptom–virus association network and heatmap (Figures 5A,C and Supplementary Table 7). Only three associations remained significant after Benjamini–Hochberg adjustment: chlorosis/yellowing was positively associated with SLCCNV (φ = 0.213, q = 0.019), stunting was positively associated with TLCCNV (φ = 0.194, q = 0.033), and mosaic was negatively associated with SLCCNV (φ = −0.182, q = 0.043) (Figure 5C and Supplementary Table 7). No other symptom–virus association, including those involving leaf curling, remained significant after correction (Figure 5C and Supplementary Table 7). These results indicate that virus–virus associations were stronger than symptom–virus associations in this symptomatic field dataset.

## 4 Discussion

### 4.1 Symptomatic pumpkin disease in Guangxi was characterized by a begomovirus-enriched virus assemblage

The present study showed that symptomatic pumpkin plants collected across Guangxi harbored a broad but highly uneven virus assemblage, in which five begomoviruses—PuYmMV, SLCPHV, TLCCNV, ToLCNDV, and SLCCNV—accounted for the dominant component of the confirmed-virus dataset. This result indicates that the current pumpkin virus situation in Guangxi is centered on a recurrent begomovirus-rich complex rather than on evenly distributed incidental virus occurrence. Because the dominant viral spectrum in a production region directly affects diagnostic targets, surveillance priorities, and breeding strategies, this pattern is epidemiologically important (Lecoq and Desbiez, 2012; Navas-Castillo et al., 2011).

Compared with cucurbit surveys reported from other regions, the Guangxi symptomatic pumpkin dataset appears more strongly enriched for begomoviruses. In Guangdong, for example, PRSV, ZTMV, ZYMV, and WSMoV were among the major viruses detected in cucurbit crops, indicating a larger contribution of RNA viruses to the regional disease complex (Li et al., 2022). Surveys from Oklahoma likewise identified cucurbit systems in which potyviruses such as PRSV-W, ZYMV, and WMV were prominent components of field infections (Khanal et al., 2021). In the Spanish Mediterranean area, recent work also documented substantial cucurbit virus diversity distributed across several virus groups rather than a clearly begomovirus-skewed pattern (López-Martín et al., 2024; Radouane et al., 2021). By contrast, begomovirus-dominated cucurbit systems with frequent mixed infections have been reported from parts of India, including Tamil Nadu and Uttar Pradesh, where begomoviruses represented a major fraction of symptomatic cucurbit infections (Nagendran et al., 2017; Kumari et al., 2021). In this context, the Guangxi pattern appears more comparable to begomovirus-dominated epidemic settings than to RNA-virus-dominated cucurbit systems.

This distinction has practical implications. Begomovirus-enriched systems may differ from potyvirus-dominated systems in their principal transmission ecology, seasonal persistence, and management priorities. Whitefly-transmitted begomoviruses are especially important in intensive vegetable systems because of their broad host ranges, efficient vector transmission, and persistence across crop and non-crop hosts (Navas-Castillo et al., 2011; Fiallo-Olivé et al., 2020; Cai et al., 2023). In addition, most viruses detected here are not restricted to pumpkin alone. Several, including ToLCNDV, TLCCNV, ZYMV, WMV, and PRSV, infect multiple cultivated cucurbits and, in some systems, additional dicot hosts or reservoir plants, creating opportunities for cross-infection among neighboring crops and non-crop hosts (Lecoq and Desbiez, 2012; Moriones et al., 2017; Li et al., 2022; López-Martín et al., 2024; Radouane et al., 2021). This host-range breadth is consistent with the repeated mixed infections observed in the Guangxi production landscape.

### 4.2 Mixed infection represented the dominant epidemiological state in symptomatic pumpkin plants

A major finding of this study was the extremely high frequency of mixed infection. Nearly all virus-positive samples carried two or more confirmed viruses, and the most common infection classes involved five or more viruses per plant. In addition, a limited number of repeated infection profiles accounted for a substantial proportion of the symptomatic dataset, with the most frequent profile consisting of the simultaneous detection of five dominant begomoviruses. These results indicate that mixed infection was the predominant epidemiological state in the surveyed pumpkin fields.

This observation is consistent with an expanding body of literature showing that mixed viral infections are common in crop systems and can influence symptom expression, virus accumulation, transmission, and disease outcome (Moreno and López-Moya, 2020; Sadras et al., 2024; Moya-Ruiz et al., 2024; Sánchez-Tovar et al., 2025). In naturally infected field plants, symptoms rarely reflect the action of a single pathogen in isolation, particularly when several viruses co-circulate within the same production environment. The high proportion of multi-virus detections observed here therefore provides a plausible explanation for the extensive symptom complexity recorded in the Guangxi pumpkin samples.

At the same time, the present data support inference about recurrent co-detection rather than direct biological interaction. Because this study was based on cross-sectional virus detection in naturally infected field samples, it cannot determine infection order, within-host facilitation, antagonism, or stable synergistic mechanisms among co-occurring viruses. The repeated joint occurrence of several begomoviruses is nevertheless notable, as shown by the strong pairwise associations among these viruses. Thus, mixed infection in Guangxi appears to have a strong and non-random structure, but mechanistic conclusions will require controlled co-inoculation studies, temporal infection assays, or quantitative within-host analyses.

The recurrent begomovirus-rich profiles observed in this study may also have longer-term evolutionary relevance. Mixed infections are well-recognized as settings in which recombination can occur more readily in geminiviruses, and experimental work has shown that recombination may arise frequently when multiple related viruses co-infect the same host (García-Andrés et al., 2007; Martin et al., 2011). Although the present study did not generate full-genome sequences from individual co-infected plants and therefore cannot demonstrate ongoing recombination, the repeated detection of multiple begomoviruses in single hosts identifies a field context in which recombination-oriented surveillance would be justified.

### 4.3 Regional heterogeneity suggests differences in local transmission ecology, but causal drivers remain unresolved

The significant regional differences detected for ZTMV, PRSV, TLCCNV, and ToLCNDV indicate that virus occurrence in Guangxi was not spatially uniform. ZTMV and PRSV were more frequent in northern Guangxi, whereas TLCCNV and ToLCNDV were more frequent in southern Guangxi. These results suggest that the pumpkin virus complex in Guangxi is structured not only by broad virus composition, but also by spatial heterogeneity in the relative prevalence of specific viruses.

Several non-exclusive mechanisms may contribute to this pattern. Regional differences in vector abundance, vector composition, cropping intensity, host availability, and inoculum continuity may all influence virus prevalence in vegetable production systems. In particular, the epidemiological importance of begomoviruses is closely tied to the biology of *Bemisia tabaci*, and previous work has shown that begomovirus transmission efficiency can vary among whitefly species or cryptic species (Fiallo-Olivé et al., 2020). Recent evidence that ToLCNDV transmission depends on cryptic-species identity within the *B. tabaci* complex provides a biologically plausible explanation for uneven regional begomovirus prevalence (Shan et al., 2025). In addition, broader phylogenomic analyses have reinforced the substantial evolutionary and biogeographic structuring within the *B. tabaci* complex, highlighting that whitefly-mediated virus pressure should not be treated as uniform across regions (Wang et al., 2025).

However, these explanations remain provisional in the context of the present study. Whitefly populations were not sampled, vector identity was not determined, and local cropping systems or reservoir hosts were not quantitatively compared among regions. Accordingly, the present results should not be interpreted as direct evidence that vector structure drove the observed regional differences. A more defensible interpretation is that the significant contrasts in virus detection are consistent with differences in local transmission ecology and inoculum pressure, but the underlying causes remain unresolved. This point is important because it prevents over-interpretation while still preserving the practical value of the regional findings. From a management perspective, the observed heterogeneity supports the need for regionally informed surveillance rather than a single uniform monitoring scheme across Guangxi.

### 4.4 Virus–virus associations were stronger than symptom–virus associations in the symptomatic field dataset

The pairwise association analyses showed that the strongest structure in the dataset was found among viruses rather than between viruses and symptoms. A dense positive co-detection module linked the dominant begomoviruses, especially PuYmMV, SLCPHV, SLCCNV, TLCCNV, and ToLCNDV, whereas only a small number of symptom–virus associations remained significant after correction for multiple testing. This contrast indicates that in symptomatic pumpkin plants from Guangxi, infection structure was defined more clearly by recurrent virus assemblages than by one-to-one correspondence between individual viruses and visible symptom categories.

This outcome is biologically plausible. In field systems where mixed infection is common, symptom expression reflects the combined effects of host genotype, tissue status, infection history, virus accumulation, and interactions among co-infecting viruses rather than the action of a single virus alone (Moreno and López-Moya, 2020; Moya-Ruiz et al., 2024; Sadras et al., 2024; Sánchez-Tovar et al., 2025). Under such conditions, symptom-based diagnosis may still be useful for identifying suspect plants in the field, but it is unlikely to provide reliable etiological resolution. The present results support exactly this interpretation: chlorosis/yellowing, deformation, blistering, mosaic, curling, and stunting were common in the symptomatic dataset, but these categories showed limited specificity for particular viruses once multiple-testing correction was applied.

The weak symptom–virus signal should also be considered in relation to the sampling design. Because the present study focused exclusively on symptomatic plants, the dataset was intentionally enriched for conspicuous disease phenotypes. Such a design is appropriate for characterizing the viral spectrum associated with field disease, but it does not capture asymptomatic infections or the full host-associated reservoir of circulating viruses. Studies in other crop systems have shown that inclusion of asymptomatic plants and nearby non-crop hosts can greatly expand the apparent virome and alter inference about epidemiological connectivity (McLeish et al., 2022; Rivarez et al., 2023). Therefore, the current symptom analyses are most informative for symptomatic field diagnosis, not for defining the total ecological distribution of these viruses in Guangxi.

### 4.5. Host-associated differences were limited and should be interpreted cautiously

The host comparison showed that none of the 11 confirmed viruses differed significantly among *C. moschata*, *C. maxima*, and *C. pepo* after multiple-testing correction. Although descriptive differences were visible for some viruses, especially among the less frequently represented host categories, the present data do not support strong claims of host-specific susceptibility. This result suggests that within the symptomatic pumpkin samples collected here, virus occurrence was shaped more strongly by the overall field infection environment than by clear host-species partitioning.

This cautious interpretation is especially important because the host groups were highly unbalanced in sample size, with *C. moschata* accounting for most of the surveyed plants and the other two species represented by much smaller numbers. Under such conditions, descriptive frequency differences can be informative as preliminary observations, but they are not sufficient to infer robust host effects. The present study therefore supports only a limited conclusion: the dominant begomovirus-rich infection pattern was not restricted to a single cultivated pumpkin species within the symptomatic dataset.

From a practical standpoint, this finding is still relevant. The absence of strong host-associated segregation implies that broad virus surveillance and diagnostic screening should not be narrowly focused on one cultivated pumpkin species. Instead, mixed begomovirus infection should be considered a shared phytosanitary concern across the main cultivated pumpkin types in Guangxi. Stronger inference regarding host preference or host-dependent infection structure would require more balanced sampling, experimental inoculation, or stratified surveys designed specifically for host comparison.

### 4.6 Implications for surveillance, breeding, and future research

An important practical implication of the present study is that pumpkin virus monitoring in Guangxi should move beyond single-virus diagnostics. Although ToLCNDV is clearly an important component of the local disease complex and has emerged as a major cucurbit pathogen in multiple regions (Moriones et al., 2017; Cai et al., 2023; Vo et al., 2025), the current dataset shows that it most often occurred within a broader begomovirus-rich assemblage rather than as an isolated agent. Recent reports of ToLCNDV in cucurbit crops in China further support the need for close surveillance, but they also indicate that its local importance should be interpreted within a dynamic and already complex cucurbit virus landscape (Gu et al., 2023; Zeng et al., 2023).

Accordingly, surveillance in Guangxi should prioritize multiplex detection strategies capable of simultaneously tracking the dominant begomoviruses together with the main RNA viruses identified in this study. Such an approach would more accurately reflect field infection conditions than single-target testing. The same logic applies to breeding and resistance evaluation. Recent genetic studies have identified molecular markers and resistance loci relevant to ToLCNDV and related begomoviruses in cucurbits, demonstrating that resistance-oriented improvement is feasible (Koeda et al., 2024; Schafleitner et al., 2024). However, the recurrent co-detection of several begomoviruses in the present dataset suggests that resistance assessment based on a single virus may underestimate the challenge posed under field conditions in Guangxi.

The present findings also point to several priorities for future work. First, broader epidemiological sampling should incorporate asymptomatic pumpkin plants, nearby cucurbit crops, weed hosts, and vector populations in order to define the wider transmission network of these viruses. Second, controlled inoculation experiments are needed to determine whether the dominant co-detection profiles identified here reflect facilitation, neutrality, or antagonism among co-infecting viruses. Third, full-genome sequencing of viruses recovered from individual mixed-infected plants would be valuable for tracking strain diversity and evaluating recombination risk within the begomovirus subset. Together, such work would extend the present molecular epidemiological baseline into a more mechanistic understanding of virus circulation and disease development in Guangxi pumpkin production systems.

Finally, the sequence-confirmation strategy used here should be interpreted conservatively. The Sanger sequences generated in this study were short diagnostic amplicons from conserved genomic regions and were used only to confirm PCR/RT-PCR product identity by BLASTn comparison. They were not submitted as independent genome records and were not used for phylogenetic analysis, because such short conserved fragments provide limited resolution for isolate-level evolutionary inference. This point is especially important for begomoviruses, for which formal species demarcation and robust phylogenetic placement are based on complete DNA-A genome sequences. Complete-genome sequencing of representative Guangxi isolates will therefore be an important direction for future taxonomic, recombination, and evolutionary analyses.

## 5 Conclusion

In summary, the present study indicates that symptomatic pumpkin disease in Guangxi is characterized by broad virus diversity, pervasive mixed infection, and a strongly recurrent begomovirus-rich co-detection structure. Regional differences were evident for several viruses, but host-associated and symptom-associated patterns were comparatively weak after correction for multiple testing. These findings support a management framework centered on multiplex diagnosis, regionally informed surveillance, and broader begomovirus-oriented resistance screening. More generally, the Guangxi system appears to represent a field setting in which mixed infection is the rule rather than the exception, and in which understanding the disease complex requires attention to virus assemblages rather than to single-virus occurrence alone.

## Data Availability

The original contributions presented in this study are included in this article/Supplementary material, further inquiries can be directed to the corresponding author.
